# Identification and Expression Analysis of MATE Genes Involved in Flavonoid Transport in Blueberry Plants

**DOI:** 10.1371/journal.pone.0118578

**Published:** 2015-03-17

**Authors:** Li Chen, Yushan Liu, Hongdi Liu, Limin Kang, Jinman Geng, Yuzhuo Gai, Yunlong Ding, Haiyue Sun, Yadong Li

**Affiliations:** College of Horticulture, Jilin Agricultural University, Changchun, Jilin, China; University of Tasmania, AUSTRALIA

## Abstract

Multidrug and toxic compound extrusion (MATE) proteins are the most recently identified family of multidrug transporters. In plants, this family is remarkably large compared to the human and bacteria counterpart, highlighting the importance of MATE proteins in this kingdom. Here 33 Unigenes annotated as MATE transporters were found in the blueberry fruit transcriptome, of which eight full-length cDNA sequences were identified and cloned. These proteins are composed of 477–517 residues, with molecular masses ~54 kDa, and theoretical isoelectric points from 5.35 to 8.41. Bioinformatics analysis predicted 10–12 putative transmembrane segments for *Vc*MATEs, and localization to the plasma membrane without an N-terminal signal peptide. All blueberry MATE proteins shared 32.1–84.4% identity, among which *Vc*MATE2, *Vc*MATE3, *Vc*MATE5, *Vc*MATE7, *Vc*MATE8, and *Vc*MATE9 were more similar to the MATE-type flavonoid transporters. Phylogenetic analysis showed *Vc*MATE2, *Vc*MATE3, *Vc*MATE5, *Vc*MATE7, *Vc*MATE8 and *Vc*MATE9 clustered with MATE-type flavonoid transporters, indicating that they might be involved in flavonoid transport. *Vc*MATE1 and *Vc*MATE4 may be involved in the transport of secondary metabolites, the detoxification of xenobiotics, or the export of toxic cations. Real-time quantitative PCR demonstrated that the expression profile of the eight *Vc*MATE genes varied spatially and temporally. Analysis of expression and anthocyanin accumulation indicated that there were some correlation between the expression profile and the accumulation of anthocyanins. These results showed *Vc*MATEs might be involved in diverse physiological functions, and anthocyanins across the membranes might be mutually maintained by MATE-type flavonoid transporters and other mechanisms. This study will enrich the MATE-based transport mechanisms of secondary metabolite, and provide a new biotechonology strategy to develop better nutritional blueberry cultivars.

## Introduction

Plants produce a large number of secondary metabolites that appear to have little function in their growth and development, but play important roles in reproduction and environmental adaptation. Secondary products are classified into three major groups: alkaloids, terpenoids, and phenolic compounds [1]. Flavonoids belong to a group of phenolic compounds and constitute one of the largest classes of secondary metabolites possessing a common three-ring chemical structure (C6–C3–C6). Predominant flavonoids forms include anthocyanins, proanthocyanidins (PAs, condensed tannins), flavonols, flavones, and flavanols. These compounds are widely distributed in different amounts according to the plant species, organ, developmental stage, and growth conditions [2]. Flavonols are thought to protect against UV-B irradiation in fruit skin [3] and function as an auxin transporter [4]. Anthocyanins have been demonstrated to give rise to the red, blue, and purple colors of many ripe fruits, vegetables, flowers, and other plant tissues or products [5], which attract frugivores and pollinators for seed dissemination and fertilization [6]. PAs in immature fruits help deter frugivores from consuming the fruit before they ripen due to their astringency and bitterness [7]. In addition, PAs are also thought to contribute to disease defense, stress resistance, and seed dormancy [8,9].

It is well known that flavonoids are synthesized along the general phenylpropanoid pathway by the activity of a cytosolic multienzyme complex that is loosely associated to the cytoplasmic face of the endoplasmic reticulum (ER) [10]. Because flavonoids with high chemical reactivity are toxic endogenous compounds, they must be removed expeditiously from the cytoplasm after synthesis and sequestered in the vacuole or cell wall. Additionally, it is also necessary for flavonoids to be sequestered in the proper compartment to prevent oxidation [11] and to perform its function as pigments. Accumulation within the appropriate compartments should be regulated in a highly sophisticated manner. Most of the structural genes and a number of regulatory genes related to the flavonoid pathway have been thoroughly described [12–14]. However, the mechanisms involved in the downstream steps of the pathway, such as transport and vacuolar accumulation, remain unclear.

In recent years, an increasing body of genetic, biochemical, and molecular biological evidence has implicated that multidrug and toxic compound extrusion (MATE) transporters are involved in flavonoid transport. The MATE family was originally discovered in prokaryotes and their function as a multiple drug efflux carrier was studied in detail. Thus far, putative MATE transporter sequences have been identified in the plant kingdoms, with some members reportedly involved in a much broader range of biological activities than previously thought (S1 Table), including protection of plant cells from inhibitory compounds [15], salicylic acid (SA)-dependent disease resistance signaling [16], aluminum tolerance [17,18], detoxification of heavy metals [19], maintenance of iron homoeostasis [20,21], alkaloids accumulation [22,23], plant development [24], and flavonoid vacuolar accumulation [25,26]. The connections between MATE transporter and flavonoid vacuolar accumulation have become topics of intensive research.

As a flavonoid/H^+^-antiporter, MATE transporters have a substrate preference for flavonoids with diverse chemical structures. The *Arabidopsis* gene TT12 acts as a transporter for the PA precursors epicatechin and catechin, while PA precursors undergo a glycosylation step prior to TT12-mediated transfer into seed coat vacuoles [2]. In vitro, TT12 exhibits an extended substrate specificity in vitro, accepting glycosylated anthocyanidin. Yeast vesicles expressing TT12 can transport anthocyanin-3-O-glucoside in the presence of MgATP, but not aglycones and epicatechin [27]. MATE1 from *Medicago truncatula*, an ortholog of TT12 expressed in yeast, preferentially transports the epicatechin 3’-*O*-glucoside, which has been proposed to be a precursor for PA biosynthesis [28,29]. Despite the high similarity between MATE1 and MATE2, the latter cannot efficiently transport PA precursors. In contrast, MATE2 shows higher transport capacity for anthocyanins and lower efficiency for other flavonoid glycosides [28,29]. MTP77 [30] and *Vv*AM1/3 [25] are all anthocyanin transporters, but *Vv*AM1/3 mediates acylated anthocyanin transport specifically rather than malvidin 3-O-glucoside or cyanidin 3-O-glucoside. However, plant MATEs have not been extensively analyzed to determine the relationship between their conformation, functional catalytic residues, and substrate preference.

Blueberry (*Vaccinium corymbosum*) is one of the most commercially significant berry crops. Blueberry fruit, which are rich in flavonoids, have received much attention due to their benefits to human health, including protection against liver injuries, significant blood pressure reduction, improved eyesight, strong anti-inflammatory and antimicrobial activities, inhibition of mutations caused by mutagens from cooked food, and suppression of proliferation of human cancer cells [31]. However, the existing researches on MATE-type flavonoid transporters were focused on model plants such as *Arabidopsis*, *M*. *truncatula*, and *Vitis vinifera*. Information regarding MATE transporters in blueberry has not been reported. A blueberry fruit transcriptome library allowed us to discover MATE transcripts as exhaustively as possible today. The objective of the present study was to clone MATE genes to provide basic data about *Vc*MATE transporters and identify candidate genes from closely related species of the family Ericaceae. Moreover, the relationship between the expression specificity of *Vc*MATEs and anthocyanin accumulation was investigated for predicting their physiological functions. This research on *Vc*MATEs participating in flavonoid transport will fill a major gap in the complete picture of blueberry metabolic pathways, and also will provide us with better strategies for plant metabolic engineering of flavonoids to improve agronomic traits and the nutritional content of blueberries.

## Materials and Methods

### Plant materials

The *Vaccinium* spp. cultivar “Patriot” grown in the blueberry germplasm repository of Jilin Agricultural University was used for this study. Representative samples were taken from six stages: flower buds and leaf buds were collected at the germination stage (2012.5.5); flowers were collected at the full-bloom stage (2012.5.15); roots, stem, and young expanding leaves were all collected from vegetative organs during the rapid growth period (2012.6.1); fruits were collected at the green fruit period (2012.6.15), color turning period (2012.7.5), and fruit maturation period (2012.7.15). The separated exocarp, sarcocarp, and seed from the blueberry fruits were immediately frozen in liquid nitrogen and stored at −80°C.

### Extraction of genomic DNA, total RNA, and cDNA synthesis

Genomic DNA was extracted from young blueberry leaves using the improved CTAB method [32]. Total RNA was isolated from 12 representative samples according to the method described in Jaakola, et al., 2001 [33]. The integrity of DNA and RNA was checked on a 1.2% agarose gel, and the purity was determined by spectrophotometry. RNA (1 μg) was used as a template for first-strand cDNA synthesis using a Superscript-II Reverse Transcriptase (TaKaRa) according to the manufacturer’s instructions.

### cDNA cloning

Candidate MATE sequences were selected from the transcriptome database of the blueberry fruit. Based on Unigenes, specific primers were designed using the Primer Premier 5 software and are listed on S2 Table. The Genome Walking Kit (TaKaRa) was used to isolate the 5′-end of Unigene27, Unigene347, and Unigene17196, while the 5′-Full RACE Kit (TaKaRa) was used to isolate the 5′-end of Unigene34286 and Unigene34357. The 3′-end of Unigene34357 was obtained with a 3′-Full RACE Core Set Ver.2.0 Kit (TaKaRa). All manipulations were conducted according to the manufacturer's protocol. The amplified fragment was sequenced and compared with other plants to confirm whether it is a MATE homolog. The full-length MATE cDNA sequences were obtained by linking the 5′ fragment, Unigene, and 3′ fragment using CExpress software. In order to ensure that the amplified segments and Unigene were within the same sequence, eight pairs of specific primers were designed in the start codon and adjacent termination codon base on linking sequences to amplify full-length cDNAs (S3 Table). In the 20 μl standard rTaq (TaKaRa) PCR, 0.4 μl of first-strand total cDNA from the mature fruit was used as a template. The thermal cycling parameters were as follows: 94°C for 3 min; 35 cycles of 94°C for 1 min, (Tm-3)°C for 1 min, and 72°C for 2 min; followed by 72°C for 10 min. All target bands were recovered and cloned into the pMD18-T vector (TaKaRa) to transform *E*.*coli* TOP10 competent cells (TIANGEN). PCR-positive colonies were sequenced using the general primers, M13F and M13R.

### Quantitative real-time PCR (qRT-PCR)

Gene-specific primers for qRT-PCR were designed by the Primer Express software and were listed in S4 Table. qRT-PCR was conducted with SYBR Premix Ex Tag (Tli RnaseH Plus) (TaKaRa) and 2 μl cDNA (diluted to 20X) as a template. Thermal cycling conditions were as follows: an initial enzyme activation of 30 s at 95°C, followed by 40 cycles of denaturation for 10 s at 95°C, annealing and extension for 20 s at 60°C, with a final melting gradient starting from 60°C and heating to 95°C for 15 s, 60°C for 1 min, and 95°C 15 s. The qRT-PCR reactions were conducted using a 7300 Fast Real Time PCR System (ABI). Primer specificity was confirmed by dissociation curves of the PCR amplification products. The mean Ct values were normalized against the reference, *V*. *myrtillus* glyceraldehyde-3-phosphate dehydrogenase (GAPDH) (NCBI accession number: AY123769.1). Since primer efficiencies were approximately equal, the expression was calculated by the 2^–ΔCt^ method. To ensure the reliability of expression results, qRT-PCR was performed for all genes on three separate cDNA preparations for each organ and fruit developmental stage. For additional replication, all templates and standards were run in triplicate.

### Identification of total anthocyanins

Total anthocyanins were identified according to the pH differential method [34]. Two dilutions of the crude blueberry extract were prepared for each sample, one with potassium chloride buffer (0.025M, pH 1.0) and the other with sodium acetate buffer (0.4 M, pH 4.5) using a predefined dilution factor. After equilibrating at room temperature for 1 h, the absorbance at 520 and 700 nm was measured by spectrophotometry. Anthocyanins content was calculated using a molar extinction coefficient for cyandin-3-glucoside of 26,900 and a molecular weight of 449.2. The quantity of anthocyanins on a fresh weight basis was expressed as mg/100g fresh weight. Cyanidin-3-glucoside was selected since it is the most common anthocyanin pigment in nature [35].

### Statistical analysis

To determine the relationship between the *VcMATE* expression level and total anthocyanin accumulation during the fruit ripening process as well as in different organs, the coefficient of determination (R^2^) was calculated with the SPSS Statistics 17.0 software. A p-value of 0.05 was considered statistically significant.

### *In silico* sequence analysis

Molecular weight and theoretical pI were predicted using the ProtParam tool (http://web.expasy.org/protparam/). Secondary structures of the eight blueberry MATE proteins were predicted by SOPMA (http://npsa-pbil.ibcp.fr/). Topological analysis for transmembrane regions was conducted using the HMMTOP program (http://www.enzim.Hu/hmmtop/index.html.). The PSORT program (http://psort.hgc.jp/form.html) was used for subcellular localization analysis. Signal peptides were predicted using SignalP 4.1 (http://www.cbs.dtu.dk/services/SignalP/). Multiple sequence alignment of amino acid sequences was performed with Clustalx1.83. Formatting of aligned sequences was accomplished using the GENEDOC program. The nonrooted neighbor-joining tree was generated with the MEGA 5.0 program.

### Accession numbers

Protein sequences in this article can be found in the *Arabidopsis* Genome Initiative or GenBank/EMBL databases with the following accession numbers: *M*. *truncatula* MATE1, ACX37118; MATE2, HM856605. *A*. *thaliana* MATE transporters TT12, NP_191462; FFT, BAE98568; FRD3, NP_001154595; ALF5, AAK21273; EDS5, AAL27003; DXT1, NP_178496 and ZRIZI,NP_564731. *Solanum lycopersicum* (tomato) MTP77, AAQ55183. *Nicotiana tabacum* (tobacco) JAT1, CAQ51477; Nt MATE1, BAF47751; Nt MATE2, BAF47752. *Hordeum vulgare* (barley) Hv ACCT1, BAF75822. *Sorghum bicolor* (sorghum) MATE transporter Sb MATE1, ABS89149; *V*. *vinifera* MATE transporters AM1 and AM3, XP_002282932 and CAO69962, respectively.

## Results

### Identification and cloning of full-length *Vc*MATE cDNA sequences

Because of the small number of available ESTs and lack of genomic information, identification of candidate MATE genes from blueberry is far behind that of other model plants. Recently, the complete of blueberry fruit transcriptome was sequenced using the Illumina RNA-Seq method, thus providing an opportunity for identification of candidate MATE transporters related to secondary metabolism. 64,312,746 raw reads longer than 75 bp were generated and made available in the Sequence Read Archive at the NCBI (http://www.ncbi.nlm.nih.gov/Traces/sra/sra.cgi) with accession number SRA046311. These relatively short reads can be effectively assembled, and a total of 34,464 All-unigenes with an average length of 735 bp were generated by de novo assembly. 8,193 differentially expressed genes (DEGs) between the exocarp and sarcocarp were categorized by molecular function, among which 516 DEGs were shown to be associated with transporter activity. Thirty-three available All-unigenes (6.4%) identified sequences encoding proteins with a significant similarity to MATE. Table 1 lists the blueberry MATE Unigenes identified in the blueberry fruit transcriptome database. Of these, 13 and 20 Unigenes were up-regulated in sarcocarp and exocarp, respectively. Among the up-regulated sarcocarp Unigenes, using TARAAPE rawreads >700, TARAAPE RPKM >10, and log_2_(TB/TA)>1 as a threshold, two sequences (Unigene27 and Unigene7401) were selected for cloning full-length cDNA sequences of *Vc*MATE. Of the up-regulated exocarp Unigenes, using TBRAAPE raw reads >700, TBRAAPE RPKM >10, and |log_2_(TB/TA)|>1 as a threshold, six sequences (containing Unigene347, Unigene2915, Unigene17196, Unigene30398, Unigene34286, and Unigene34357) were selected. When the eight Unigenes were compared by Blaxtn to Genbank data, we found that Unigene2915, Unigene7401, and Unigene30398 covered the complete coding region, while the remaining Unigenes were missing sequence from the 3'-end, 5'-end, or both. Then, the corresponding full-length cDNA sequences of the five partial sequences were obtained by Genome walking and RACE technology. The eight full-length sequences of blueberry MATE transporters identified in this study were named *VcMATE*1, *VcMATE*2, *VcMATE*3, *VcMATE*4, *VcMATE*5, *VcMATE*7, *VcMATE*8, and *VcMATE*9 as shown in Table 2, which also lists the accession number of their complete cDNA and the closest corresponding *Arabidopsis* genes.

**Table 1:** Unigenes with a significant similarity to MATE in the Illumina/Solexa sequencing library of blueberry.

Unigene	Nr-ID	Gene length	TARAAPE rawreads	TBRAAPE rawreads	TARAAPE RPKM	TBRAAPE RPKM	log_2_(TB/TA)	Up/Down
Unigene27_All	NP_200058.1	1462	1573	853	71.9099	31.7664	−1.1787	Down
Unigene 347_All	ADO22709.1	1496	873	3212	39.0023	116.8988	1.5836	Up
Unigene 1047_All	NP_194643.1	681	606	226	59.4748	18.0687	−1.7188	Down
Unigene 2475_All	NP_194643.1	388	571	196	98.3584	27.5037	−1.8384	Down
Unigene 2915_All	AAD46034.1	1878	95	1450	3.3809	42.0377	3.6362	Up
Unigene 4510_All	AAK53040.1	592	226	99	25.5149	9.105	−1.4866	Down
Unigene 4079_All	ACC60274.1	2199	1677	1720	50.97	42.5862	−0.2593	Down
Unigene 4546_All	ADO22709.1	510	58	149	7.6009	15.9068	1.0654	Up
Unigene 5999_All	NP_001057687.1	660	327	126	33.114	10.3942	−1.6717	Down
Unigene 7401_All	XP_002890090.1	1819	773	399	28.4023	11.9428	−1.2499	Down
Unigene 10025_All	NP_194034.1	212	138	61	43.5061	15.6661	−1.4736	Down
Unigene 13574_All	AAK53040.1	1136	608	263	35.7711	12.605	−1.5048	Down
Unigene 14339_All	ADO22709.1	389	48	122	8.2471	17.0756	1.05	Up
Unigene 16852_All	NP_177270.1	910	1809	2027	132.8631	121.277	−0.1316	Down
Unigene 17196_All	NP_194294.2	1780	280	1267	10.5134	38.7545	1.8821	Up
Unigene 17373_All	NP_001043893.1	227	230	308	67.7188	73.8739	0.1255	Up
Unigene 19867_All	NP_200058.1	587	69	73	7.8563	6.71	−0.2145	Down
Unigene 20486_All	NP_001043893.1	227	41	119	12.0716	28.5422	1.2415	Up
Unigene 23136_All	XP_002278724.1	372	25	7	4.4916	1.0245	−2.1323	Down
Unigene 25244_All	NP_194643.1	270	80	18	19.8031	3.6297	−2.4478	Down
Unigene 28730_All	AAK53040.1	368	0	41	0	6.066	12.5665	Up
Unigene 28970_All	NP_566730.1	338	1	40	0.1977	6.4433	5.0264	Up
Unigene 29667_All	BAF47751.1	437	0	57	0	7.1017	12.7939	Up
Unigene 29812_All	XP_002278724.1	540	4	51	0.4951	5.1421	3.3766	Up
Unigene 30026_All	NP_189291.1	1507	38	743	1.6853	12.3922	2.8784	Up
Unigene 30398_All	NP_187012.2	1689	1	959	0.0396	30.914	9.6085	Up
Unigene 31039_All	BAF47752.1	201	9	150	2.9926	40.6314	3.7631	Up
Unigene 31994_All	XP_002278724.1	227	2	36	0.5889	8.6346	3.874	Up
Unigene 32737_All	AAK53040.1	261	1	0.2561	19	3.9635	3.952	Up
Unigene 33021_All	ADO22711.1	282	18	28	0.5889	8.6346	3.874	Up
Unigene 33638_All	ABA96342.2	342	0	19	0	3.0248	11.5626	Up
Unigene 34286_All	NP_187012.2	642	38	3144	3.956	266.6328	6.0747	Up
Unigene 34357_All	BAF47752.1	786	34	2005	2.8911	138.8858	5.5861	Up

Note:TARAAPE and TBRAAPE rawreads represent the gene abundance of the sarcocarp and exocarp, respectively, TARAAPE RPKM (TA) and TBRAAPE RPKM (TB) is used for the expression level of sarcocarp and exocarp, respectively.

**Table 2:** Putative MATE transporters from blueberry and their closest Arabidopsis homologs.

Unigene	Gene	Accession number	Arabidopsis closest homolog	Arabidopsis AGI gene
Unigene27	*VcMATE*1	KF875433	AtDTX16	AT5g52450
Unigene347	*VcMATE*2	KF831422	AtDTX41/AtTT12	AT3g59030
Unigene2915	*VcMATE*3	KF875434	AtDTX33	AT1g47530
Unigene7401	*VcMATE*4	KF875435	AtDTX12	AT1g15170
Unigene17196	*VcMATE*5	KF875436	AtDTX34	AT4g00350
Unigene30398	*VcMATE*7	KF875438	AtDTX24	AT3g03620
Unigene34286	*VcMATE*8	KF875439	AtDTX24	AT3g03620
Unigene34357	*VcMATE*9	KF875440	AtDTX40	AT3g21690

### Analysis of deduced MATE proteins in blueberry

**Fig 1:**
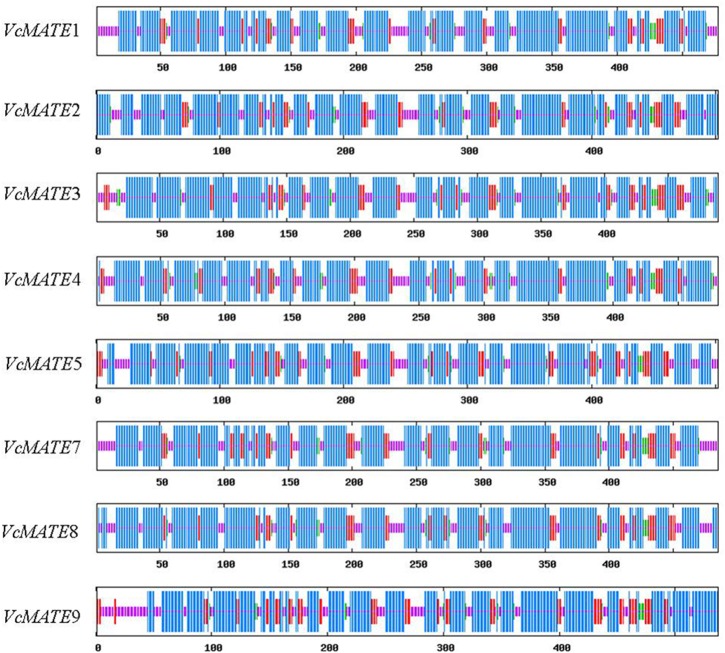
Secondary structures of the 8 blueberry MATE proteins predicted by SOPMA. The α-helix, extended strand, β-turn, and random coil are denoted as the longest (blue), medium long (dark red), short (green), and the shortest (pink) vertical bars, respectively.

**Fig 2:**
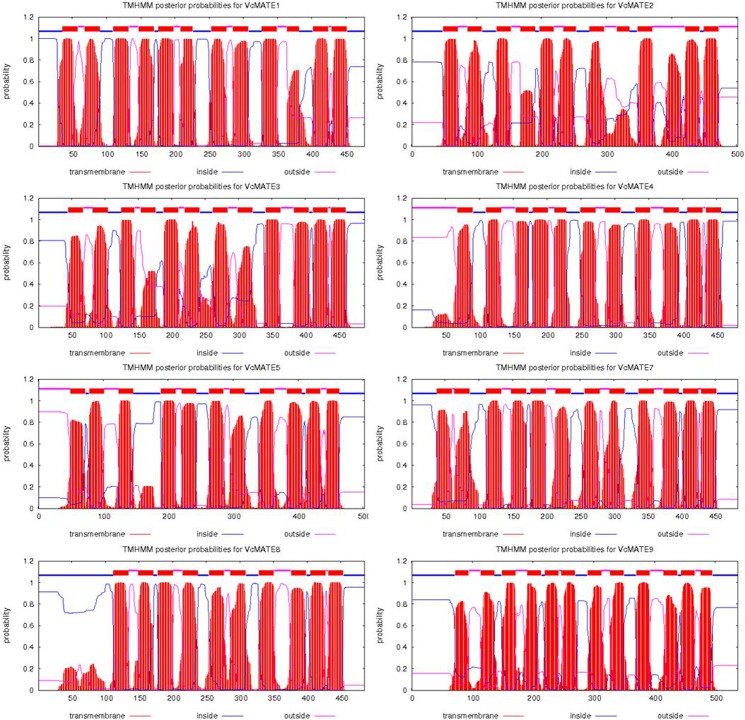
Topological analysis of VcMATEs. The membrane-spanning domains of VcMATEs were determined using the TMHMM2 program.

As presented in Table 3, *Vc*MATE cDNA encoded proteins are composed of 477–537 residues, with predicted molecular masses around 54 kDa, theoretical isoelectric points from 4.89 to 8.41, aliphatic indices between 113.88 and 126.29, and grand average of hydropathicity around 0.7. All deduced *Vc*MATE proteins were predicted as stable because their instability index is smaller than 40. When analyzed by SOPMA, the secondary structures of blueberry MATEs were predicted to be mainly composed of alpha helices (60.34–68.13%) interspersed with random coils (18.13–22.35%), extended strands (10.36–14.53%), and beta turns (2.39–4.09%) (Fig. 1). For each protein, there were more than 15 distinct alpha helices dispersed along the length of the protein. The prediction of transmembrane domains using HMMTOP suggested that there were 10–12 putative transmembrane (TM) segments in *Vc*MATEs (Fig. 2). Prediction of the subcellular localization of *Vc*MATEs was performed using the PSORT program. The results showed that the eight transporters were all localized to the plasma membrane (Table 3). Proteins are usually guided to their target membranes by signal peptides, but there were no signal peptides in the N-terminus according to SignalP. It is possible that signal peptides do not have strictly conserved amino acid sequences and are thus sometimes difficult to identify.

**Table 3:** Characteristics of the VcMATE cDNA deduced proteins.

	Number of amino acids	Formula	Molecular weight (kDa)	Theoretical pI	Instability index	Aliphatic index	Grand average of hydropathicity,GRAVY	Predicted protein localization
*Vc*MATE1	477	C_2362_H_3731_N_593_O_630_S_35_	51.64	8.41	25.72	113.88	0.675	plasma membrane
*Vc*MATE2	502	C_2539_H_3961_N_603_O_675_S_25_	54.54	5.57	28.05	126.29	0.842	plasma membrane
*Vc*MATE3	489	C_2418_H_3785_N_587_O_648_S_25_	52.25	5.35	29.70	119.86	0.820	plasma membrane
*Vc*MATE4	481	C_2377_H_3768_N_586_O_650_S_30_	51.92	6.87	29.00	116.96	0.701	plasma membrane
*Vc*MATE5	502	C_2527_H_3930_N_608_O_677_S_24_	54.43	5.70	25.87	121.95	0.759	Plasma membrane
*Vc*MATE7	484	C_2472_H_3849_N_593_O_647_S_26_	53.06	6.00	38.61	124.11	0.791	plasma membrane
*Vc*MATE8	485	C_2500_H_3875_N_589_O_662_S_23_	53.48	4.89	33.05	125.67	0.786	plasma membrane

### *Vc*MATE multiple sequence comparison

**Fig 3:**
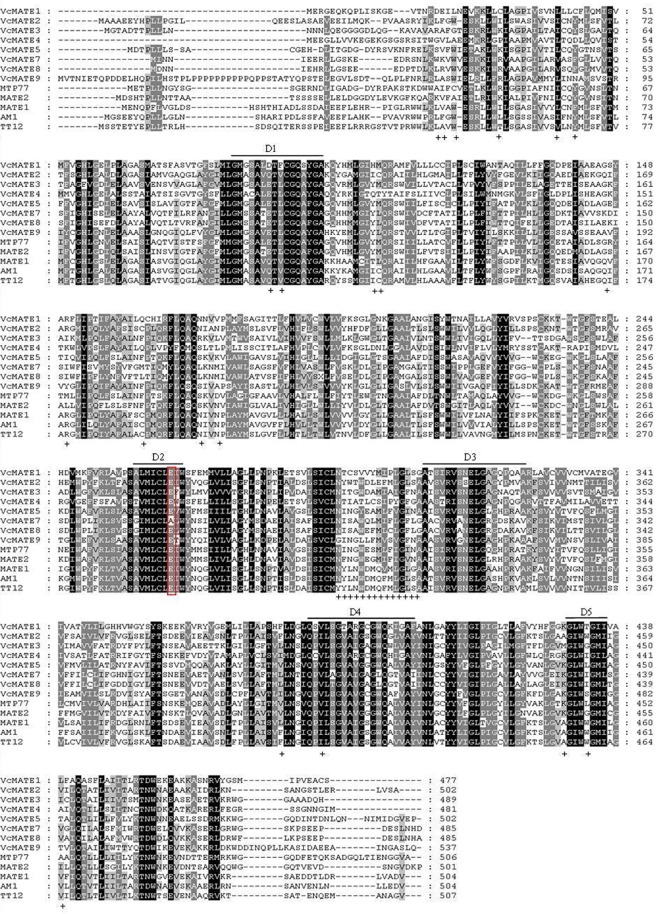
Multiple sequence alignment of deduced amino acid sequences of VcMATEs with selected MATE transporter orthologs. Protein sequence alignment was performed using Clustalx. Formatting of aligned sequences was accomplished with the box shade program. Amino acids that are identical in all 8 proteins are highlighted in black and conservative substitutions are highlighted in gray. Alignment of all 56 *Arabidopsis* MATE proteins resulted in five short stretches containing conserved amino acids, which are highlighted by thin lines above the alignment and noted as D1–5. The residue E290 (TT12), constituting the cation-binding site in the pore, is highlighted by a red box.

Multiple sequence alignment was conducted between the eight deduced *Vc*MATE amino acid sequences and some representative MATE-type flavonoid transporters in other plant species (Fig. 3). The eight *Vc*MATEs share 32.1–84.4% amino acid sequence identity amongst each other. The most striking finding in the sequence analysis was that *Vc*MATE2 was more similar to the MATE-type flavonoid transporters, the *V*. *vinifera* MATE transporters AM1 (75.4% identity), the *Arabidopsis* TT12 (70.8% identity), *Medicago* MATE1 (70.4% identity) and MATE2 (40.8% identity), and tomato MTP77 (40.6% identity), than other *Vc*MATEs. At less than 40%, *Vc*MATE1 and *Vc*MATE4 shared the lowest identity with known MATE-type flavonoid transporters. Five short stretches containing conserved amino acids in all 56 *Arabidopsis* MATE proteins also appeared to be particularly conserved in blueberry, which are highlighted by bars below the alignment and noted as D1-D5. The amino acid residue E290, which is critical to the proper functioning of *At*TT12 [36], is also conserved in all *Vc*MATEs except *Vc*MATE7. Moreover, only *Vc*MATE2 contains all the TT12-specific residues, such as KL_(46–47)_, W_50_, A_61_, Q_116_, V_118_, IC_132–133_ Q_173_, R_177_, C_190_, N_200_, N_203_, G_297_, YYLNWDMQFMLGLS _(319–332)_, F_408_, I_413_, KTS _(449–451)_, A_456_, W_460_, and V466 in *Arabidopsis* [37].

### Phylogenetic analysis of *Vc*MATEs

**Fig 4:**
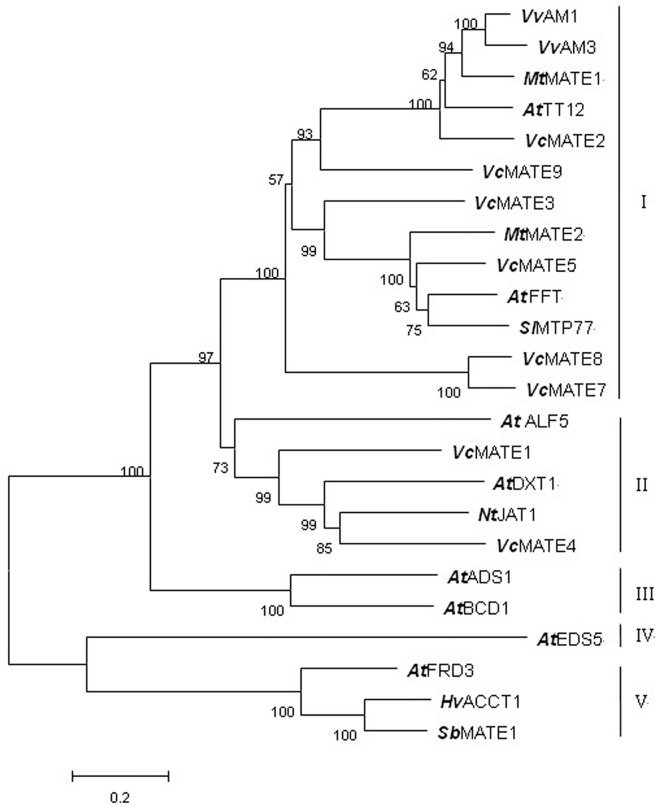
Phylogenetic tree of MATE transporters. Protein sequences of the characterized MATE transporters from *Arabidopsis*, TT12, FFT, ALF5, DXT1, ADS1, ZRIZI, EDS5, and FRD3; M. truncatula MATE1 and MATE2; sorghum Sb MATE1; grapevine AM1 and AM3; tomato MTP77; barley Hv AACT1; as well as tobacco JAT1 were aligned with Clustalx, and the nonrooted neighbor-joining tree was generated using the MEGA 5.0 program. Numbers at branch points indicate bootstrap support. The numbers above and below branches indicate bootstrap support percentages based on 1000 replicates.

To further predict and distinguish the function of eight *Vc*MATEs, phylogenetic analysis was performed between 18 known plant MATE protein sequences of different types and the putative *Vc*MATEs. Phylogenetic analysis of these proteins revealed the presence of five distinct clusters (clusters I, II, III, IV, and V in Fig. 4), implying that the five clusters might differ functionally in some respect. The first clade contained *Vc*MATE2, *Vc*MATE3, *Vc*MATE5, *Vc*MATE7, *Vc*MATE8, *Vc*MATE9, and some known MATE-type flavonoid transporters. In addition, we found three subclades within the first clade: *Mt*MATE1, *At*TT12, *Vv*AM1/3, and *Vc*MATE2 were grouped into one subclade; *Vc*MATE3 and *Vc*MATE5 together with *Mt*MATE2, *At*FFT, and *Ml* MTP77 formed another subclade; while *Vc*MATE7 and *Vc*MATE8 were in a separate subclade. *Vc*MATE1, *Vc*MATE4, *Arabidopsis* toxin exporter DTX1, and ALF5, together with nicotine transporter JAT1 were grouped into the second clade. ADS1, which constitutively accumulates reactive oxygen intermediates (ROIs) to negatively regulate plant disease resistance, and BCD1, which enhances organ initiation, were grouped together in the third clade. The salicylic acid (SA) transporter EDS5 formed an individual clade. Three plasma membrane-localized citrate exporters, *Sb*MATE from sorghum, *Hv*AACT1 from barley, and FRD3 from *Arabidopsis*, were grouped into the last clade, which was much further removed from the eight *Vc*MATE transporters. Transporter substrate specificity typically correlates with phylogeny, and hence such analyses provide a credible foundation for making functional predictions. This phylogenetic analysis suggested that *Vc*MATE2, *Vc*MATE3, *Vc*MATE5, *Vc*MATE7, *Vc*MATE8, and *Vc*MATE9 might function as flavonoid transporters to participate in the transport of anthocyanins, PAs, or flavonols, while *Vc*MATE1 and *Vc*MATE4 might be involved in the detoxification of xenobiotics or export of toxic cations.

### Accumulation of anthocyanins in different organs and fruit developmental stages

**Fig 5:**
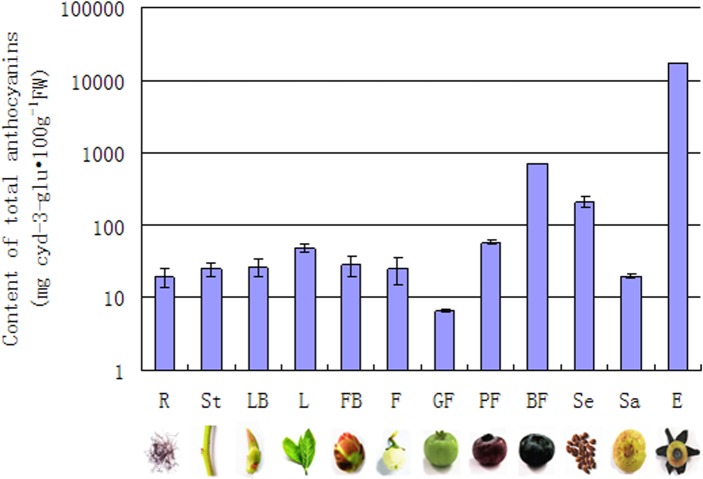
Anthocyanin content in different organs and developmental stages of fruit. The following 12 samples were analyzed: R-roots, S-stem, FB-flora buds, F-flowers, LB-leaf buds, L-young leaves, GF-green fruit, PF-pink fruit, BF-blue fruit, E-exocarp, Sa-sarcocarp, and Se-seed. Values represent the average ± SD of three biological replicates.

Blueberries are well known for their nutritional and beneficial health effects owing to their high anthocyanins content. The concentration of anthocyanins in different organs and fruit developmental stage was determined using a pH differential method. The result showed that anthocyanins were weekly detected in the vegetative organs. In fruit development, exocarp color will change from mostly green to partially pink, finally blue-purple. In the green fruit stages, anthocyanins were detected at low levels. In accordance with their deep coloration, the anthocyanins content increased, reaching the maximum in mature berries. Moreover, the anthocyanins content of exocarp in blue fruit was observed to be significantly higher than sarcocarp. Meanwhile, anthocyanins also accumulated in substantial quantities in seed. (Fig. 5)

### Blueberry organ and fruit developmental profiling of *VcMATE* transcripts

**Fig 6:**
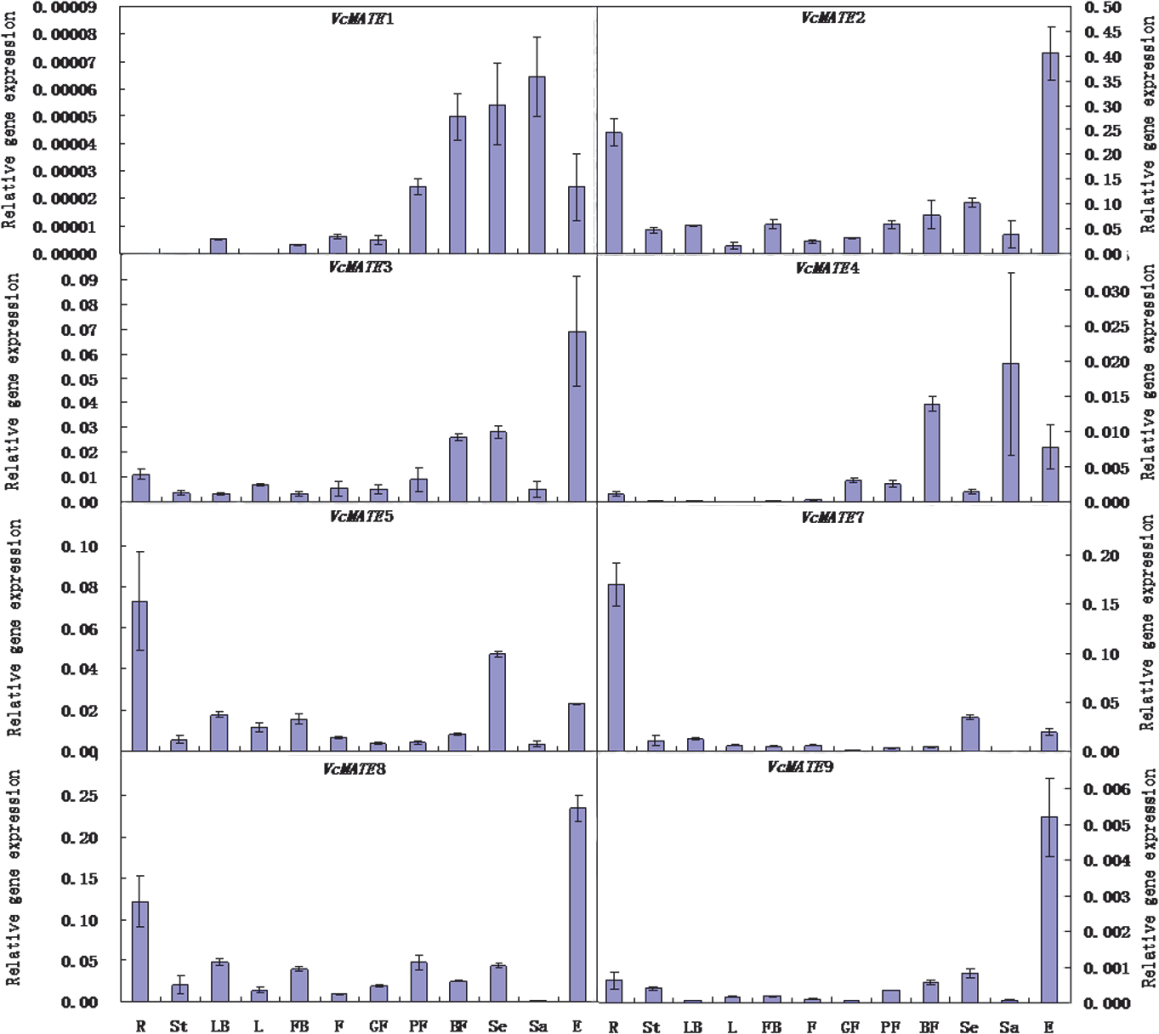
Relative gene expression levels of MATE-encoding genes in various blueberry organs and different developmental stages of fruit. Total RNA was isolated from roots (R), stem(S), flora buds (FB), flowers (F), leaf buds (LB), young leaves (YL), green fruit (GF), pink fruit (PF), blue fruit (BF), exocarp (E), sarcocarp (Sa), and seed (Se). Gene expression was normalized with *Vm*GAPDH. All data represent the mean of three replicates with error bars indicating SD.

Anthocyanins content varied in the different organs, developmental stages of fruit, and parts of the mature fruit. This was reflected in the apparent concerted activation of anthocyanin biosynthesis gene expression. Next, we examined the expression pattern of *VcMATE* genes in accordance with anthocyanin accumulation. Quantitative real-time PCR was performed on eight MATE genes throughout fruit development and different organs (Fig. 6). The expression level of *VcMATE*2 was the highest among the eight genes. *VcMATE*2 transcript was found most strongly expressed in anthocyanin-rich exocarp, and followed by root. During fruit developmental stages, expression was low in green fruit stage and then increased after fruit coloring. The coefficient of determination (R^2^) between *VcMATE*2 expression and total anthocyanins accumulation was 0.852. *VcMATE*3, *VcMATE*8 and *VcMATE*9 followed a similar expression pattern with *VcMATE*2, and R^2^ values between the expression and anthocyanin accumulation were calculated as 0.913, 0.876 and 0.987, respectively. Contrast to *VcMATE*2, the transcript abundance of *VcMATE*8 in mature fruit was lower than pink fruit, while transcript abundance of *VcMATE*3 and *VcMATE*9 in root was not much higher than other organs. *VcMATE*5 and *VcMATE*7 were predominantly expressed in root. Moreover, both of *VcMATE*5 and *VcMATE*7 present at higher transcript abundance in seed than exocarp. Transcript level of *VcMATE*1 and *VcMATE*4 were much weaker than other genes. The relevance between expression and total anthocyanins accumulation was low, even negative correlation. Furthermore, no *VcMATE*1 transcripts were detected in root, steam, and leaf. There was another obvious difference between *VcMATE*1, *VcMATE*4 and other *VcMATE* genes, the highest expression site for the two genes was sarcocarp rather than exocarp. The expression level of sarcocarp and exocarp of all *VcMATE* genes obtained by qRT-PCR analysis were consistent with the expression found by *in silico* analysis. Gene expression patterns of MATE family genes varied in the different organs and fruit developmental stages, suggesting that different expression patterns reflect different functions. *VcMATE*2, *VcMATE*3, *VcMATE*5, *VcMATE*7, *VcMATE*8, and *VcMATE*9 may function as flavonoid transporters due to their relatively high expression level and correlation between expression profile and anthocyanin accumulation. *VcMATE*1 and *VcMATE*4 may be involved in other functions.

## Discussion

The MATE protein is an emerging member of the multidrug transporter superfamily that includes the major facilitator superfamily (MFS), the small multidrug resistance (SMR) family, the resistance nodulation cell division (RND) family, and the ATP-binding cassette (ABC) family [38]. This family is widely distributed in all living organisms from prokaryotes to eukaryotes [39]. As a secondary active transporter, MATE is thought to couple transport of their target molecules across a membrane with an electrochemical gradient of H^+^ or Na^+^ ions, requiring the action of a plasma membrane P-type H^+^-ATPase, a vacuolar V-type H^+^-ATPase, or a vacuolar H^+^-pyrophosphatase [40]. In contrast to the relatively small number of MATE genes found in bacterial and animal species, this gene family has undergone a remarkable expansion in plants, with 58 MATEs in *Arabidopsis*, 57 in *V*. *vinifera*, 58 in *Populus trichocarpa*, and 38 in *Zea mays* and *Brachypodium distachyon* (ARAMEMNON plant membrane protein database). This finding highlights the importance of MATE proteins in this kingdom. The identification of genes specifying traits of interest through a candidate gene approach was hindered due to a lack of blueberry genomic information; however, the fruit transcriptome database makes this possible now. There were 33 Unigenes annotated as MATE efflux family proteins. In addition, eight differentially expressed *VcMATE*s between the exocarp and sarcocarp with higher gene abundance and expression level were obtained. We speculate that they may play a vital role in the detoxification of endogenous secondary metabolites and xenobiotics.

### Structure of *Vc*MATE transporter is related to its function

The length of proteins in the MATE family ranges from ~400 to ~700 amino acids, with most members consisting of 400–550 residues [41]. The yeast proteins are larger (up to about 700 residues), whereas the archaeal proteins are generally smaller. A large transporter size is characteristic of the eukaryotic domain, while a small size is characteristic of the archaeal domain [42]. The eight *Vc*MATE transporters with 477–537 amino acids conform to the basic characteristics of MATE family members. MATE precursors were hypothesized to be complex carbohydrate exporters in prokaryotes with six TMs which underwent internal duplication to generate a 12 TM protein [43]. It is interesting to note that *Vc*MATEs have 10–12 TMs predicted by the HMMTOP program rather than the usual 12 TMs that characterize the MATE family. MATE transporters possessing more or less than 12 TMs also have been reported in some species. For example, 8–13 TMs in the third cluster of genes was found in *Arabidopsis* MATE proteins [19], 9–11 in EDS5 [16], and 14 in the FRD3 protein [44]. The mammalian MATEs are generally predicted to have a 13th TM; however, TM13 has been confirmed to have little impact on ligand binding and the functional core of MATE1 consists of 12 (not 13) TMs [45]. Studies have shown that the few proteins possessing more than the usual 12 TM were derived from an internal gene duplication event, and that the “extra” regions are presumably nonessential for transport function [43]. However, other proteins have fewer than 12 TMs, possibly because the prediction of topology was performed using a different program with different computer methods. According to the TMHMM program, these data are only predictions based on computer analysis of protein sequences; therefore, they must be considered cautiously.

There were no consensus sequences to be conserved across all MATE members, which shared approximately 40% sequence similarity. However, we found that some TM domains were much more conserved than other sites, and some inter-TM linkers were remarkably conserved. Human MATE1 is a representative secondary structure for MATE-type transporters. Multiple sequence alignment has indicated that highly conserved regions are located in the vicinity of transmembrane helix 1 (TM1) and TM7, within the extracellular loops connecting TM1 with TM2 and TM7 with TM8, in cytoplasmic loops linking TM2 with TM3 and TM8 with TM9, and in loops connecting TM4 with TM5 and TM10 with TM11 [46]. Chai, et al. found two tandemly arranged MatE/NorM domains in plant TT12 proteins spanning from TM1 to TM5 and from TM7 to TM11 [37]. Conserved protein domains indicate they may be functional sites of MATE transporters. Quite possibly MATE proteins transport “cargo” via a pore maintained by the TM helices. It is necessary to maintain the secondary structure and topology of all the TM domains for normal MATE function, but conservation of some TMs and inter-TM linkers might be crucial for formation of the “pore” [37]. Alignment of all 56 *Arabidopsis* MATE proteins resulted in five short stretches (D1–D5) containing conserved amino acids, which also appear to be conserved in *Vc*MATEs. Thus, we think that the five domains consisting of conserved amino acids may guide sequence-based identification of MATE transporters. Furthermore, E290, a conserved amino acid critical for transport and vacuolar targeting in *Arabidopsis*, was also present in *Vc*MATEs. The E290 was hypothesized to exert a cation-binding function similar to E255 in NorM, which faces the internal cavity and is in proximity to the cavity-located cation [47]. However, until now, the structure-function relationships of plant MATEs has yet to be investigated. Thus, our results are in accordance with the hypothesis that E264 in *Vc*MATE1, E285 in *Vc*MATE2, E276 in *Vc*MATE3, E267 in *Vc*MATE4, E276 in *Vc*MATE5, E265 in *Vc*MATE8, and E308 in *Vc*MATE9 may be involved in cation binding in *Vc*MATEs.

Although MATE transporters are distributed widely across all kingdoms of living organisms, only model bacterial and human MATEs have been characterized in detail. An outward-facing conformation with two portals open to the outer leaflet of the membrane was reported in NorM from *Vibrio cholerae* [47]. Then, the structures of *Pyrococcus furiosus* reveal that a drug extrusion mechanism in which the protonation of Asp41 on the N-terminal lobe induces bending of TM1, which in turn collapses the N-lobe cavity, thereby extruding the substrate drug to the extracellular space [48]. In this study, we failed to predict information on the tertiary structure of the eight *Vc*MATE proteins using the SWISS-MODEL because sequence identity was too low, implying that they might well have a unique tertiary structure distinct from that of the characterized NorM and *Pyrococcus furiosus*. However, all MATE proteins share similar transmembrane topologies. Further research is needed to determine whether flavonoid transporters have the opposite structure or a similar mechanism of action with efflux proteins.

### Subcellular localization of MATE transporters

The subcellular localization of a transporter is crucial for its function. The plant MATE proteins characterized so far are localized to either the vacuolar membranes or plasma membranes, which are two primary sites for iron uptake and sequestration [49]. The substrate is transported out of the cells in exchange for the H^+^- influx when the transporter is localized to the plasma membrane. However, when the transporter is localized to the vacuolar membrane, it functions as an uptake transporter because the cytosolic pH (7.2–7.5) is higher than that of the vacuolar lumen, which is generally 5.5 [22]. Some MATE-type flavonoid transporters have been shown to localize in the tonoplast. It is necessary for most secondary metabolites to be delivered into vacuoles because most of them are toxic to the plant itself. Their vacuolar compartmentalization may improve the efficiency of their production and avoid harmful effects in the cells [50]. Unique among plant MATE transporters identified so far, ZRZ is localized to the membrane of a small organelle, possibly the mitochondria, suggesting that ZRZ may be involved in providing a complex network of communication about a leaf-borne signal that determines the rate of organ initiation [24]. Recently, BCD1 (previously named ZRZ) protein was observed to localize to the Golgi complex, suggesting that BCD1 is associated with excretion of excess iron, which would be produced in chlorotic cells under osmotic stress conditions and senescing leaf cells [51]. In this research, subcellular localization of *Vc*MATEs has been predicted using the bioinformatics tool PSORT. The results revealed that all deduced proteins localize to the plasma membrane. However, *Vc*MATE2, *Vc*MATE3, *Vc*MATE5, *Vc*MATE7, *Vc*MATE8, and *Vc*MATE9 were phylogenetically related to the known flavonoid transporters MATE1, MATE2, TT12, AM1, and AM3, all of which appear to be localized to the vacuolar membrane on the basis of GFP fusion imaging. Our prediction about targeting information is different with vacuolar localization of MATE-type flavonoid transporter. This may result from some membrane transporters lacking discernible targeting information for bioinformatics tools to recognize. Therefore, the localization of *Vc*MATEs requires further validation.

### Functional diversity of *Vc*MATE members

In recent years, plant MATEs have been genetically identified, characterized, and shown to have particular physiological functions. Transporter substrate specificity typically correlates with phylogeny. Hence such analyses provide a credible foundation for making functional predictions. In order to gain insight into the putative role played by *Vc*MATE, phylogenetic analysis was studied between *Vc*MATEs and other known MATEs. Clustering analysis showed *Vc*MATE2, *Vc*MATE3, *Vc*MATE5, *Vc*MATE7, *Vc*MATE8, and *Vc*MATE9 were clustered together with some MATE-type flavonoid transporters. Although *Vv*AM1/3, *Mt*MATE1/2, *At*FFT, *Ml*MTP77, and *At*TT12 belong to MATE-type flavonoid transporter, no common substrate has been reported to the best of our knowledge. Generally, flavonoids are transported in the form of glycosides. *Mt*MATE1 is a functional ortholog of *At*TT12, and both of them transport E3'G with higher affinity and velocity than Cy3G [28,29]. Anthocyanins depositing in vacuoles are largely present in acylated forms. *Vv*AM1/3, *Mt*MATE2, and *Ml*MTP77 are anthocyanin transporters, with *Vv*AM1/3 involved specifically in transporting p-coumaroyl-acylated anthocyanidin glucosides (Gomez *et al*., 2009), while *Mt*MATE2 specifically transports malonylated flavonoids. Two major types of acylation, aromatic acylation (such as addition of a p-coumaroyl group) and aliphatic acylation (such as addition of a malonyl group), have been suggested to have different physiological functions. Aromatic acylation enhances the color of anthocyanins, whereas aliphatic acylation (usually malonylation) may stabilize flavonoids and increase the resistance of flavonoid glucoside malonates to enzymatic degradation [52,53]. It is important to note that flavonoid composition is different in each plant species, such that the transport activity of the *Vc*MATE transporters could be determined by transport studies. *Vc*MATE1 and *Vc*MATE4 clustered with some multidrug efflux transporters in another group. *At*ALF5 is expressed strongly in the root epidermis, and loss of ALF5 function results in increased sensitivity of the root to several compounds, including a contaminant of commercial agar [15]. *At*DTX1 shows relatively broad substrate specificity, and confers norfloxacin, berberine, and cadmium tolerance when expressed in *Escherichia coli* [19]. Nt-JAT1 plays an important role in nicotine translocation, and is responsible for unloading of alkaloids in the aerial parts and deposition in the vacuoles [22,23]. Thus, we conclude that *Vc*MATE1 and *Vc*MATE4 may be involved in transport of alkaloids and detoxification of xenobiotics or toxic cations. It is interesting to find members in the same or different cluster that play the same or opposite role. Although the eight *Vc*MATEs were not present in the other three clusters, we cannot exclude the possibility of other functions for other *Vc*MATE transporters.

### Expression patterns response to physiological processes

The eight *VcMATE* genes investigated in this study have different spatial and temporal expression patterns, possibly because of differences in transport substrates or the complex and widespread accumulation of (iso)flavonoid compounds in blueberry. *VcMATE*5 and *VcMATE*7 exhibit similar expression patterns with *Mt*MATE2, which is strongly expressed in roots [28,29]. Malonylated flavonoid glycosides are abundant in root, and *Mt*MATE2 preferentially transports malonylated flavonoids. Meanwhile, the two genes also appear to be highly expressed in seed of mature fruit. This is similar to *At*TT12 and *Mt*MATE1, which are transcribed mainly during the early stages of silique and young pod development after fertilization, especially in developing seeds [15]. The PAs found in the seed coat consist essentially of epicatechin units [54,55], and *At*TT12 and *Mt*MATE1 facilitate vacuolar uptake of epicatechin 3′-O-glucoside for PAs biosynthesis in *Arabidopsis* and *Medicago*. Expression levels of both genes did not increase with gradual deepening of the exocarp color until fruit maturation, possibly because PAs synthesis occurred primarily early in fruit development. There is competition between PAs and anthocyanin biosynthesis. Anthocyanin synthesis takes place rapidly following ripening initiation, primarily in the exocarp, which is likewise reflected in a concerted activation of anthocyanin transport gene expression. Correlation between transcript abundance of *VcMATE*2, *VcMATE*3, *VcMATE*8, and *VcMATE*9 with anthocyanin accumulation suggests that the four *VcMATE* gene products may be responsible for anthocyanidins vacuolar accumulation in the blueberry fruit. The expression patterns of *VcMATE*2, *VcMATE*3, *VcMATE*8, and *VcMATE*9 in blueberry fruit, which were also similar to that of *Vv*AM1/3, parallels the expression patterns of five previously identified key enzyme-encoding genes associated with anthocyanin biosynthetic genes in blueberry, including Unigene938 (phenylalanine ammonia-lyase, PAL), Unigene23486 (chalcone synthase, CHS), Unigene7743 (flavonoid 3-hydroxylase, F3H), Unigene8103 (dihydroflavonol 4-reductase, DFR), and Unigene26381 (anthocyanidin synthase, ANS) [56]. *VcMATE*1 and *VcMATE*4 not only had the weakest transcript level, but also the transcript patterns were different from the other six *VcMATE* genes. What's more, they were most strongly expressed in sarcocarp instead of exocarp, root, or seed, suggesting that they may be involved in other physiological processes.

The flavonoids are products of phenylpropanoid metabolism constituting more than 10,000 structural variants known [57]. Anthocyanins constitute a major flavonoid group. After synthesis on ER, anthocyanins are then subjected to various modifications, such as methylation, acylation, hydroxylation, and glycosylation, yielding a wide variety of derivatives. These metabolites across the membranes in blueberry may be maintained by diverse mechanisms. Another secondary active transporter, bilirubin transporter bilitranslocase (BTL), primary transporter ATP-binding cassette (ABC) transporters, different forms of anthocyanin pigment-containing bodies the in vesicular transport (VT) model, and glutathione S-transferases (GSTs) supporting the ligandin transporter (LT) model, have all been demonstrated to be responsible for flavonoids vacuolar deposition in some plants. Thus, MATE transporters represent one route from the site of flavonoid biosynthesis towards the vacuole and other subcellular compartments in blueberry. Different mechanisms may cooperate with each other to form a highly sophisticated transport network. Therefore, no perfect correlation between expression pattern of *VcMATE* gene and accumulation of total anthocyanins was presented in this paper. It is interesting to clarify the relationship between preferred substrates and transport mechanism. In conclusion, the present study have set a foundation for further studies on functions of *VcMATE* genes, and may provide guidance for selection of target genes to improve agronomic traits and fruit quality of blueberry.

## Supporting Information

S1 Table Properties of plant MATE-type transporters. **Modified from Yazaki et al, 2008**. *TT12-transparent testa; ALF5-aberrant lateral root formation; EDS5–enhanced disease susceptibility; FRD3–ferric reductase defective; FFT–flower flavonoid transporter; ADS1-Activated Disease Susceptibility; JAT1-jasmonate-inducible alkaloid transporter 1; FRDL1-FRD3 like 1; BCD1-bush and chlorotic dwarf n.d. not determined, PM-plasma membrane, Vac-vacuole, TMA-tetramethylammonium, PVP-polyvinylpyrrolidone, EtBr—ethidium bromide.
(XLS)

S2 Table Primers used for cloning the *Vc*MATEs. (XLS)

S3 Table Primers used for amplify of full-length cDNA. (XLS)

S4 Table Primers used for real-time quantitative RT-PCR experiments. (XLS)
